# Hyaluronic Acid Coating Reduces the Leakage of Melittin Encapsulated in Liposomes and Increases Targeted Delivery to Melanoma Cells

**DOI:** 10.3390/pharmaceutics13081235

**Published:** 2021-08-11

**Authors:** Yanyan Li, Shuyao Ruan, Zhi Wang, Nianping Feng, Yongtai Zhang

**Affiliations:** Department of Pharmaceutics, Shanghai University of Traditional Chinese Medicine, Shanghai 201203, China; LYY4248@shtrhospital.com (Y.L.); 15990101229@163.com (S.R.); wangzhi908@163.com (Z.W.)

**Keywords:** nanoparticle, active targeting, anti-tumor, nanomedicine, prolonged circulation

## Abstract

Melittin is a promising antitumor substance; however, it is a nonspecific cytolytic peptide, which limits its clinical application. In this study, melittin liposomes (Mel-Lip) and hyaluronic acid (HA)-modified Mel-Lip (Mel-HA-Lip) were designed to reduce the toxicity and increase the anti-tumor effects of melittin. The optimal preparation procedure was evaluated using a uniform design based on the single factor method, and the concentration of HA was determined based on the cellular uptake of coumarin 6 labeled HA-Lip. Liposomes and HA-modified liposomes were evaluated in vitro by assessing cytotoxicity, cellular uptake, and release behavior. Liposomes prepared in the optimum formulation improved stability, with a particle size of 132.7 ± 1.55 nm, zeta potential of −11.5 ± 1.51 mV, entrapment efficiency of 86.25 ± 1.28%, and drug-loading efficiency of 3.91 ± 0.49%. Cellular uptake tests revealed that the uptake of nanoparticles significantly increased with HA modification, suggesting that HA modification enhanced the internalization of liposomes within cells, which was consistent with the results of the cytotoxicity analysis. Furthermore, in vitro release experiments showed that Mel-HA-Lip possessed a stronger sustained-release effect compared with Mel-Lip. The results of this experiment provide insight into the potential tumor-targeting effects of melittin.

## 1. Introduction

In recent years, there have been approximately four million new cases of cancer and three million deaths due to cancer in China each year. In 2020, 19.3 million new cases of cancer were reported globally. This number is expected to exceed 28.4 million in 2040, representing an increase of about 47%, particularly in developing countries [1,2]. Although chemotherapy plays a significant role in existing anticancer regimens, its clinical effects are limited by side effects and the development of multidrug resistance following repeat administration. Thus, in the field of cancer research, there is an urgent need for the development of highly effective antitumor therapies, which improve patient outcomes [3,4,5].

Melittin is a cationic amphiphilic peptide with 26 amino acid residues extracted from venom. It confers strong biological and pharmacological activity, including antibacterial properties, radio-resistance, inhibition of platelet aggregation, antiarthritic, and anti-tumor activity [6,7]. It is soluble in methanol (about 20 mg/mL) [8], easily soluble in water (over 250 mg/mL), and accounts for approximately 40–50% of the dry weight of bee venom [9]. Among these properties, the anti-tumor effects of melittin are of particular interest to researchers [10,11]. A study found that melittin can self-crosslink in an aqueous solution to form a spiral tetramer. Due to the amphiphilic properties resulting from the polar and nonpolar amino acids on the surface of the spiral structure, melittin can easily fuse and destroy the natural or synthetic phospholipid bilayer, resulting in cell lysis, which accounts for its effects against tumor cells [12,13]. In contrast, melittin can induce apoptosis in tumor cells. Melittin was found to induce autophagy, alter the expression of apoptotic proteins in cells, and promote the apoptosis of tumor cells through the mitochondrial apoptosis pathway. In addition, melittin can inhibit the invasion and metastasis of tumor cells and is involved in immune regulation [14,15].

However, melittin is a nonspecific cytolytic peptide that lyses all prokaryotic and eukaryotic cells [16]. Furthermore, the anti-tumor application of melittin is hindered by its hemolysis, non-specificity, and ease of degradation. Mao et al. prepared poloxamer 188-coated nanoliposomes for the treatment of vascular irritation, inflammation, and allergic reaction in mice, and to enhance their inhibitory activities on hepatocellular carcinoma (HCC) cells [17]. In addition, melittin-loaded lipid nanoparticles modified with polyethyleneglycol have also demonstrated prolonged circulation in the blood and lower aggregation ability [18]. Therefore, the construction of a nanodelivery system for melittin represents a new strategy for reducing the side effects of melittin and enhancing its potential clinical application.

Liposomes are vesicles formed by phospholipids and cholesterol with a self-assembled biomembrane structure. Liposomes can encapsulate both water- and fat-soluble drugs, and have been investigated as drug-delivery systems for anti-tumor therapies [19]. The addition of cholesterol to the phospholipid membrane has been reported to restrain the liposomal vesicle leakage induced by melittin, and decreased melittin’s binding to the liposomal membrane [20].

Hyaluronic acid (HA) is a natural glycosaminoglycan and the main component of the extracellular matrix. It possesses good biocompatibility, biodegradability, and non-immunogenicity [21,22,23]. HA modification forms a hydrogel network structure on the surface of nanoparticles, which decreases drug leakage and prolongs the circulation time of nanomedicines in vivo. HA and its derivatives bind to specific receptors located in the cell membrane, including the CD44, receptor for HA-mediated mot (RHAMM), and the HA receptor for endocytosis (HARE) [24]. CD44 is a widely distributed cell-surface glycoprotein and the most systematically studied cell-surface receptor for HA. CD44 mediates transmembrane transportation through specific binding with the HA ligand to transport drugs into cells. In addition, CD44 is highly expressed on the surface of various malignant tumor cells, including melanoma, and ovarian and breast cancers [25,26]. The specific binding between HA and its derivatives and CD44 can be used to target the delivery of anti-tumor drugs to tumor cells with high CD44 expression [27,28]. Thus, the construction of an active targeting drug-delivery system for HA-modified melittin-loaded liposomes (Mel-HA-Lip) is a method for improving drug targeting, enhancing the inhibitory effect on tumor cells, decreasing drug dosage, and reducing adverse reactions.

**Figure 1:**
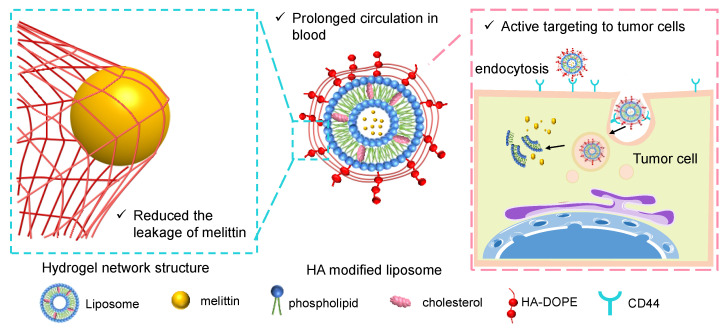
A schematic diagram of Mel-HA-Lip inhibiting the leakage of encapsulated melittin and enhancing drug delivery to tumor cells.

In this study, liposomes were used as the injection delivery carrier of melittin, and the stability of the preparation was improved by adjusting the prescription dosage of cholesterol. In addition, liposomes were modified with HA via dioleoyl phosphoethanolamine (DOPE)-conjugated HA (HA-DOPE) with covalent bonds inserted into the liposome membrane. The gel layer formed on the surface of the liposome by HA further reduced the leakage caused by the transmembrane function of melittin. Additionally, with the aid of specific binding between HA and CD44, which is highly expressed on the surface of melanoma cells (B16F10), the drug can be targeted for delivery to melanoma, thereby attenuating toxicity and enhancing anti-tumor activity (Figure 1).

## 2. Materials and Methods

### 2.1. Materials and Regents

Phosphoethanolamine-N-maleimide (polyethylene glycol)-2000 (DSPE-PEG2000), DOPE, and 1,2-diacyl-*sn*-glycero-3-phosphocholine (HSPC) were obtained from AVT Pharmaceutical Technology Co., Ltd. (Shanghai, China). Coumarin 6 (C6) and 3-(4,5-Dimethyl-2-thiazolyl)-2,5-diphenyl-2H-tetrazolium bromide (MTT) were obtained from Sigma-Aldrich (St. Louis, MO, USA) and Meilun Biotechnology Co., Ltd. (Dalian, China), respectively. Melittin was purchased from Peptide Co., Ltd. (Hangzhou, China). HA (molecular weight: 21 KDa) was purchased from Freda Pharmaceutical Group Co., Ltd. (Ji’nan, China). N-3-Dimethylaminopropyl)-N’-ethylcarbodiimide-hydrochloride (EDC) and N-Hydroxysuccinimide (NHS) were purchased from Aladdin. Phosphate-buffered saline (PBS, pH 7.4) was obtained from Dingguo Biotechnology Co., Ltd. (Shanghai, China). Cholesterol and all other chemicals were of analytical reagent grade and were purchased from Sinopharm Chemical Reagent (Shanghai, China).

### 2.2. Cell Line

B16F10 cells were purchased from the typical culture committee of the Chinese Academy of Sciences. The cells were cultured in Dulbecco’s modified Eagle medium (DMEM) supplemented with 10% fetal bovine serum (FBS) and 1% penicillin-streptomycin at 37 °C under a humidified atmosphere containing 5% CO_2_.

### 2.3. Melittin Detection

Preparations obtained from different formulations were assayed by high-performance liquid chromatography (Shimazu LC-2010AHT, Shimazu, Japan) with a DIKMA Platisil ODS column (4.6 × 250 mm, 5 μm) to detect melittin. The mobile phase was acetonitrile: 0.1% trifluoroacetic acid (40:60 *v*/*v*) with a column temperature of 25 °C. The detection wavelength for melittin and flow velocity were set at λ = 220 nm and 1 mL/min, respectively. The standard curve equation was obtained by linear regression of the peak area A to concentration C. The results showed that the regression equation in the concentration range of 25–250 μg/mL was C = 14,330 A − 312,803, with a correlation coefficient of 0.9999.

### 2.4. Preparation and Optimization of Melittin-Loaded Liposomes (Mel-Lip)

#### 2.4.1. Preparation of Mel-Lip

The nanovesicles were formulated via the reverse-phase evaporation method as follows: phospholipids and cholesterol were dissolved in an appropriate ratio in a mixed solution of chloroform and methanol (3:1, *v*/*v*) [29]. Then, melittin dissolved in PBS (pH 7.4) was added to the above mixture to obtain a concentration ratio of the organic phase to the aqueous phase of 5:1. After 5 min of ultrasonication (SB-5200D, Ningbo Scientz Biotechnology Co., Ltd., Ningbo, China), a homogeneous mixture of emulsions was formed. The emulsion in the eggplant flask was evaporated at 37 °C with rotation at 30 rpm under vacuum to remove organic solvents. PBS was added for dispersing the self-assembled phospholipid vesicles at 40 °C and 100 rpm for 1 h. Finally, the preparation was transferred to a centrifuge tube, followed by ultrasound at 52 W for 20 min in an ice bath (JY92-II, Ningbo Scientz Biotechnology Co., Ltd., Ningbo, China).

#### 2.4.2. Optimization of Mel-Lip

Single-factor experiments were performed, which investigated different package methods, various pH values of PBS, phospholipid concentration, ultrasonic power, drug concentration, and the ratio of drug to lipid, to obtain the optimal formulation using particle size, potential, polydispersity index (PDI), drug loading (DL), and encapsulation efficiency (EE) as evaluation indexes.

In addition to the reverse-phase evaporation method mentioned above, another preparation method was thin-film dispersion, as follows: phospholipids and cholesterol were weighed and dissolved in a solution of chloroform and methanol (*v*/*v* 2:1). Next, the mixture was evaporated under a vacuum at 37 °C with 30 rpm rotation to form a film. The PBS-dissolved melittin was added to hydrate the film by incubating at 40 °C and 100 rpm for 1 h. Liposomes were obtained by ultrasonication for 20 min at 52 W in an ice water bath.

Based on the results of the single-factor experiments, uniform design experiments selected melittin concentration and drug-to-lipid ratio as independent variables, with particle size, encapsulation rate, and drug loading rate as indexes. The complete design consisted of five experimental points.

### 2.5. Synthesis of HA-DOPE

As described in the previous report [30], 26 mg HA was added to 10 mL distilled water (0.1 mol/L hydrochloric acid solution to adjust the pH to 4.0) and stirred in a magnetic stirrer for 24 h to ensure complete swelling. Then, 12 mg EDC and 6.8 mg NHS were dissolved in the HA solution (adjusted to pH 4.0) with a water-bath temperature of 37 °C and 300 rpm rotation for 2 h. Then, 360 μL of 1 mg/mL DOPE in ethanol solution was thoroughly mixed with HA solution (pH adjusted to 8.6, with 0.1 mol/L sodium hydroxide solution), and the mixture was continuously stirred at 300 rpm at 37 °C for 24 h. The reaction mixture was then dialyzed (with a molecular weight cut off 3500) at 37 °C and 300 rpm for 48 h using distilled water as the dialysate. The dialysate was changed every 4–8 h to remove the remaining reactants and by-products. The final product was freeze-dried and stored at −20 °C.

### 2.6. Preparation of Mel-HA-Lip

HA-DOPE was dissolved in PBS (0.5 mg/mL) for 2 h, then was used for dispersing the self-assembled phospholipid vesicles as described above to obtain the Mel-HA-Lip. The concentration of HA-DOPE was optimized using the cellular uptake capacity as an investigation index.

### 2.7. Particle Size Distribution and Zeta Potential of Liposomes

The liposomes were diluted with deionized water and their particle size distribution and zeta potential were measured using a Malvern particle sizing system (Nano ZS 90, Malvern Panalytical, Malvern, UK). Measurements were performed in triplicate for each sample.

### 2.8. DL and EE of Liposomes

The EE and DL of liposomes were determined by the ultrafiltration-centrifugation method [31], as follows: 100 μL liposomes were centrifuged in an ultrafiltration centrifuge tube (molecular weight cut-off of 30 K) at 13,201× *g* (Minispin, Eppendorf, Hamburg, Germany) until all the dispersion media were filtered. The filter cake was washed twice with 100 µL pure water each time. The concentration of melittin in the filtrate was detected by HPLC. Simultaneously, the liposomes were dissolved in methanol (10 times, *v*/*v*) and ultrasonicated for 30 min. After centrifugation at 13,201× *g* for 10 min, the concentration of melittin was determined using HPLC. EE and DL were calculated using the following equations: EE = (W_t_ − W_y_)/W_t_ × 100%, DL = (W_t_ − W_y_)/W_n_ × 100%. Where, W_y_ is the amount of free melittin in the prepared sample, W_t_ is the total amount of melittin in the prepared sample, and W_n_ is the total mass of the liposomes, including melittin, phospholipids, and cholesterol.

### 2.9. Transmission Electron Microscopy (TEM)

The preparations were diluted with deionized water and then dripped slowly on a copper mesh. Thirty minutes later, dry filter paper was used to remove excess liquid. Finally, the preparations negatively stained with uranyl acetate for 30 s were observed under a transmission electron microscope (TEM; JEM-2100, JEOL Co. Ltd., Tokyo, Japan).

### 2.10. Drug Release In Vitro

One milliliter of liposomal dispersion (melittin concentration: 1 mg/mL) was loaded into a dialysis bag (with a molecular weight cut off of 14,000 ± 2000). Both ends of the dialysis bag were clamped and immersed in 15 mL dissolution media (PBS at pH 7.4) in a 50 mL centrifuge tube acting at 37 °C and 100 rpm rotational speed in a constant temperature oscillator with a UDT-818 Series Tester and SCR-DL auto-sampler/DSC-800 System Controller instrument (Logan Instruments Corp., Somerset, NJ, USA), then sampled at predetermined time points. Samples were dissolved in 9 times (*v*/*v*) methanol, and the concentration of Mel was determined by HPLC.

### 2.11. Cellular Uptake

C6 was used as a fluorescent probe, dissolved in methanol, and loaded into various nanovesicles as described above. The final C6 content in the preparation was 20 μg/mL. The C6 content was determined based on the fluorescence intensity measured from the Spark^TM^ 10 M multimode microplate reader (Tecan Group Ltd., Männedorf, Switzerland). With the fluorescence intensity (I) and concentration (C) of C6 as dependent and independent variables, respectively, the fitted linear regression equation was: I = 4402 C + 2512, in the concentration range of 1–7 μg/mL.

A total of 3 × 10^5^ B16F10 cells per well in the logarithmic phase were seeded into six-well plates and cultured for 24 h. The culture medium was changed to a medium containing C6-labeled liposomes (C6-Lip) and HA-modified liposomes (C6-HA-Lip) (with a C6 concentration of 1 µg/mL) without FBS after washing twice with PBS. After 1.5 h of incubation, the medium was discarded, and cells were washed three times with PBS. The cells were digested by adding pancreatin and separated by centrifugation (1000× *g*, 3 min). The cells were resuspended in PBS and transferred to a flow tube. The fluorescence intensity was measured using a flow cytometer (FACS-Canto, Becton, Dickinson and Company, Franklin Lakes, NJ, USA) and cells in the blank culture medium were used as a control. Each group was tested in triplicates. These procedures were performed under light exposure.

### 2.12. Cellular Cytotoxicity

Cell viability was evaluated by the reduction in MTT assay. Briefly, B16F10 cells (5 × 10^3^/well) in the logarithmic phase were inoculated into 96-well plates and incubated in a water-saturated atmosphere of 5% CO_2_ at 37 °C. Twenty-four hours later, a concentration series of preparations including free drugs, blank liposomes (Lip), blank HA-lip (HA-Lip), Mel-Lip, and Mel-HA-Lip were diluted in incomplete medium without FBS and replaced with the original medium for a further 24 h. Each concentration was tested in triplicate. Next, 10 μL MTT was added to each well, and cells were incubated for 4 h. All liquid was then removed, and 100 μL dimethyl sulfoxide was added and mixed. The optical density (OD) value of each well was measured based on the absorbance at 527 nm using a microplate reader. The cell viability in each group was calculated with Equation (1):

(1) $$\mathrm{Cell}\;\mathrm{viability}\;\left(\%\right)=\frac{{\mathrm{OD}}_{p}-{\mathrm{OD}}_{b}}{{\mathrm{OD}}_{c}-{\mathrm{OD}}_{b}}\times 100\%$$

where, OD_p_ is the optical density of preparations in experiments, OD_b_ is the optical density of the blank group without cells, and OD_c_ is the optical density of blank cells as a control group.

### 2.13. Statistical Analysis

The results are expressed as the mean ± standard deviation. Statistical analyses were performed using Student’s *t*-tests in SPSS software (v 13.0; IBM, Co., Armonk, NY, USA). *p*-values < 0.05 were considered statistically significant.

## 3. Results

### 3.1. Preparation of Mel-Lip

**Figure 2:**
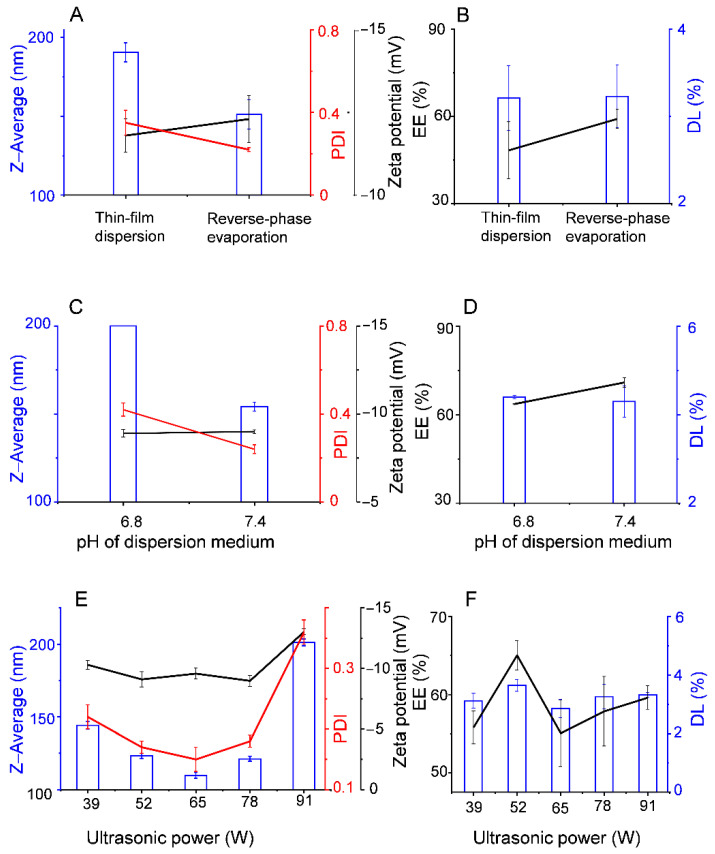
Effect of different factors during the preparation process on the characteristics of Mel-Lip: preparation method (**A**,**B**), pH of PBS (**C**,**D**), ultrasonic power (**E**,**F**). (*n* = 3).

Among the liposome membrane materials selected in this current work, DSPE-PEG2000 and HSPC have been used in commercial products (e.g., Doxil^®^, Titusville, NJ, USA). By coating hydrophilic PEG in the DSPE-PEG2000 molecules on the surface of the liposomes, the clearance of the nanocarriers by the mononuclear phagocyte system is avoided and, therefore, supports prolonged blood residence times. In addition, DSPE-PEG2000, HSPC, and DOPE molecules all contain double-long carbon chains with high saturation. The bilayers they form usually have high rigidity and low permeability, which enhances the stability of the liposomes. To study the influence of different preparation methods on the characteristics of Mel-Lip, all other preparation parameters were controlled at the same level. Figure 2A,B shows the effect of the preparation in different directions. Liposomes prepared by the reverse-phase evaporation method had a smaller particle size and PDI, as well as a larger absolute zeta potential value and EE compared with those prepared by the thin-film dispersion method.

The preparations were performed with PBS at different pH values (pH 7.4, 6.8), while the other factors remained unchanged. Figure 2C,D shows the effects of pH on the characteristics of Mel-Lip. Preparations obtained using dispersion medium at pH 7.4 had a higher EE, smaller particle size, and more even distribution compared with those obtained at pH 6.8.

Under the same conditions, ultrasonic power played a significant role in the characteristics of Mel-Lip. Figure 2E showed that the particle size first decreased and then increased with increasing ultrasonic power. Further, the Mel-Lip had the smallest particle size when the ultrasonic power was 65 W. However, it was also noted that an ultrasonic power of 52 W was the definite point, which resulted in the highest EE and DL (Figure 2F). Notably, EE is a leading index used to measure the quality of liposome preparations. Therefore, an ultrasonic power of 52 W was found to be the optimal choice, and was able to achieve the maximum EE and relatively smaller particle size.

**Figure 3:**
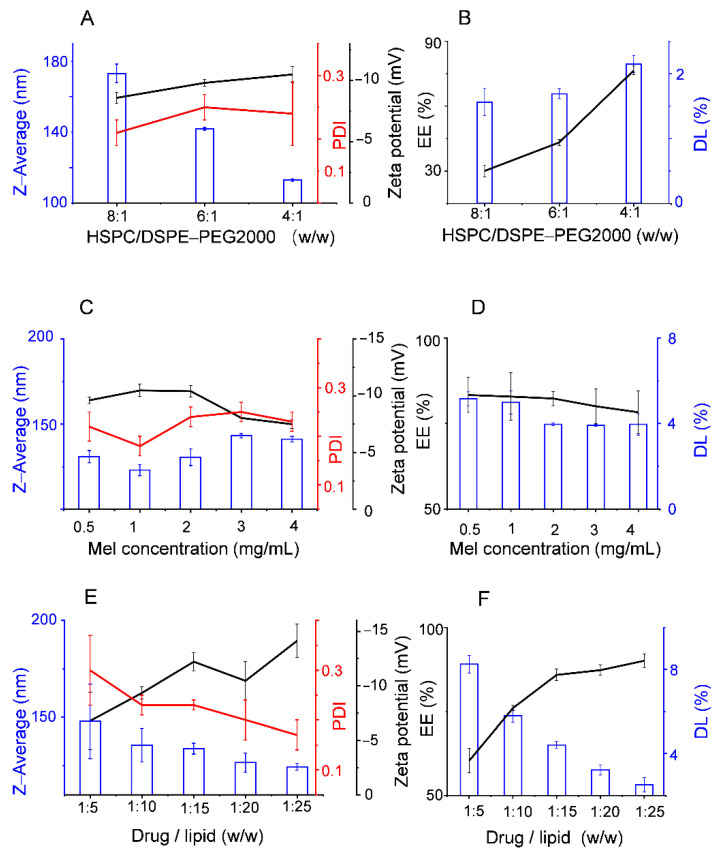
Effect of different factors during the preparation process on the characteristics of Mel-Lip: HSPC to DSPE-PEG2000 mash ratio (**A**,**B**), concentration of melittin (**C**,**D**), drug to lipid mass ratio (**E**,**F**). (*n* = 3).

The influence of phospholipid concentration was shown in Figure 3A,B. The EE and absolute potential value increased, and particle size decreased when the HSPC/DSPE-PEG2000 mash ratio ranged from 8:1 to 4:1. The results indicated that an HSPC/DSPE-PEG2000 weight ratio of 4:1 was conducive to a smaller particle size and higher EE and DL.

Figure 3C,D shows the impact of melittin concentration on Mel-Lip. The drug concentration mainly concerned the EE and DL of the preparations. The DL decreased sharply when the melittin concentration reached 2 mg/mL. Considering various factors, the use of melittin at concentrations of 0.5 or 1 mg/mL generated liposomes with smaller particle sizes, larger absolute potential value, and higher EE and DL.

The effect of the drug/lipid weight ratio is shown in Figure 3E,F. The EE of liposomes was affected by the concentration of lipids and drugs. Within the range of the experiment, EE increased and DL decreased as the concentration of lipid increased. A drug to lipid ratio of 1:15 was found to be optimal for obtaining a relatively satisfactory and eligible EE and DL.

Based on the results of the single-factor experiment, five experimental runs were performed to optimize the drug concentration (x_1_) and drug/lipid weight ratio (x_2_) in a uniform design experiment to maximize DL and EE, and minimize the particle size of the Mel-Lip (Table 1). A nonlinear regression equation was obtained by SPSS software, as follows: mean size (nm) = 129.609 − 3.32x_1_ − 0.04x_2_; EE (%) = 87.974 − 6.932x_1_ − 0.012x_2_; DL (%) = 3.252 + 0.997x_1_ + 0.002x_2_. The optimal preparation was achieved using the Visual Basic program grid method, with a drug concentration of 1 mg/mL and a drug/lipid ratio of 1:15.

To validate the model equation, three parallel experiments were performed under the optimal conditions. The experimental values obtained for particle size, zeta potential, EE, and DL are shown in Table 2. The mean particle size was about 132.7 ± 1.55 nm with a PDI of 0.23 ± 0.01, which indicated that the nanovesicles comprised an even distribution of particle size. The EE was larger than 80%, indicating that the Mel-Lip represented a promising preparation with potential clinical application.

### 3.2. Preparation of HA-Mel-Lip

**Figure 4:**
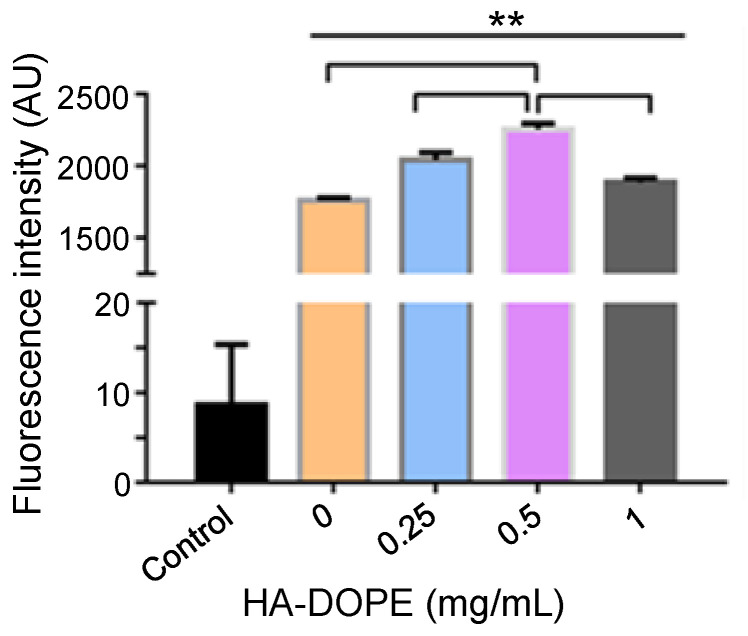
Mean fluorescence intensity of B16F10 cells following treatment with C6-HA-Lip with different HA-DOPE concentrations. (Comparison between groups, ** *p* < 0.01; *n* = 3).

The concentration of HA was determined by cellular uptake. As shown in Figure 4, cellular uptake improved with increasing HA concentration, while an overabundance of HA hindered uptake. This was due to competitive adhesion between free HA-DOPE and HA-Lip at the cell membrane, thereby decreasing the amount of HA-Lip internalization. The data indicated that the optimal concentration of HA-DOPE in the preparation was 0.5 mg/mL.

**Figure 5:**
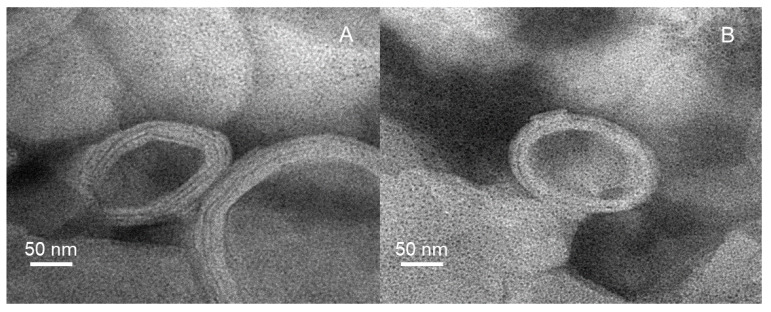
TEM images of Mel-Lip (**A**) and Mel-HA-Lip (**B**).

Based on the optimal formulation, the Mel-HA-Lip was prepared and characterized (Table 2). Compared with Mel-Lip, the average size of Mel-HA-Lip showed no significant difference (*p* > 0.05), while EE markedly increased (*p* < 0.01), suggesting that HA modification enhanced the stability of the nanovesicles. In addition, Figure 5 showed the characteristic morphological changes of the liposomes after modification. The Mel-Lip presented spherical or ellipsoid morphology with obvious lipid bilayers, while the Mel-HA-Lip (drug concentration: 250 μg/mL, HA-DOPE concentration: 0.5 mg/mL) became more rounded under TEM.

### 3.3. Drug Release In Vitro

**Figure 6:**
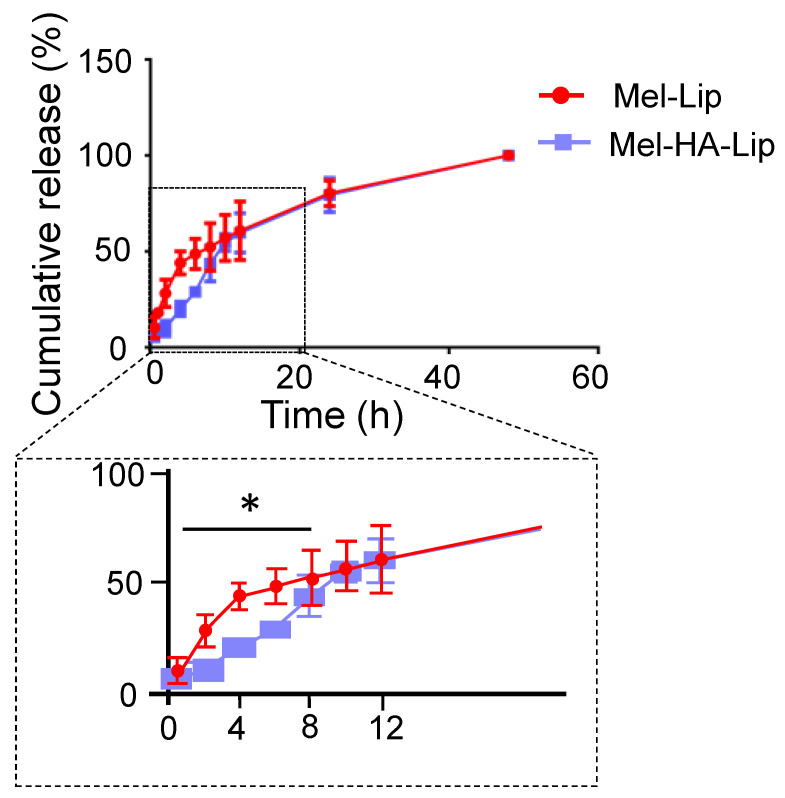
In vitro release profiles of melittin released from Mel-Lip and Mel-HA-Lip. (Mel-Lip compared with Mel-HA-Lip, * *p* < 0.05; *n* = 3).

The experimental results are presented in Figure 6. The cumulative release of Mel-Lip and Mel-HA-Lip differed significantly in the first 12 h. Following HA modification, liposomes were able to slow the release of the encapsulated drug. However, there was no significant difference in the amount of cumulative release after 12 h. The quantity released cumulatively over 48 h was close to 100% in each case.

### 3.4. In Vitro Evaluation of Anti-Tumor Activity

**Figure 7:**
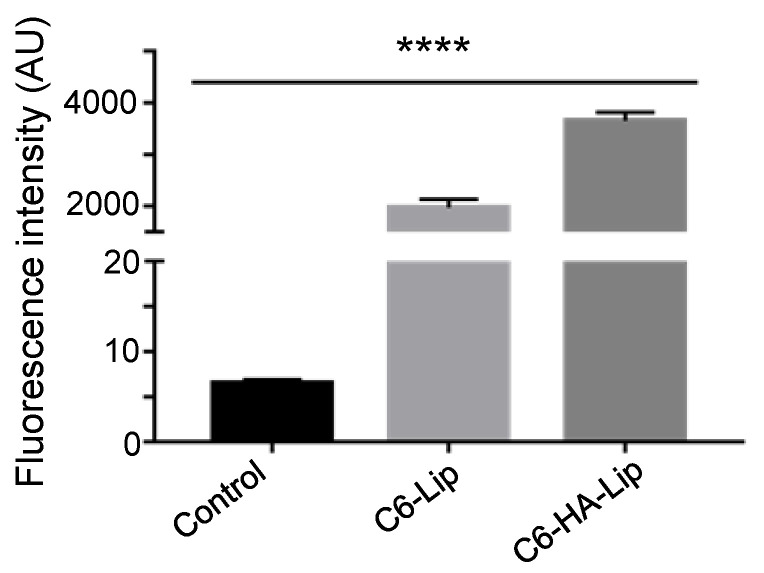
Mean fluorescence intensity of B16F10 cells following treatment with C6-Lip and C6-HA-Lip. (Comparison between groups, **** *p* < 0.0001; *n* = 3).

Compared with liposomes, the HA-modified liposomes presented higher cellular uptake, indicating that the intracellular delivery of liposomes to B16F10 was promoted following HA modification (Figure 7).

**Figure 8:**
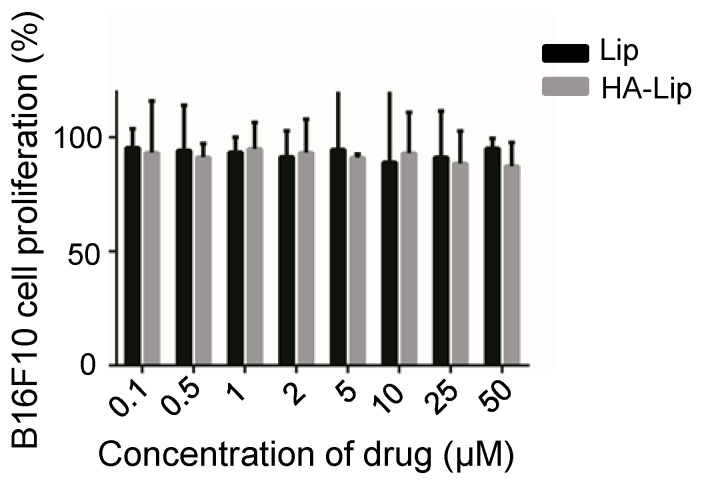
Cytotoxicity of the blank vector. (*n* = 3).

**Figure 9:**
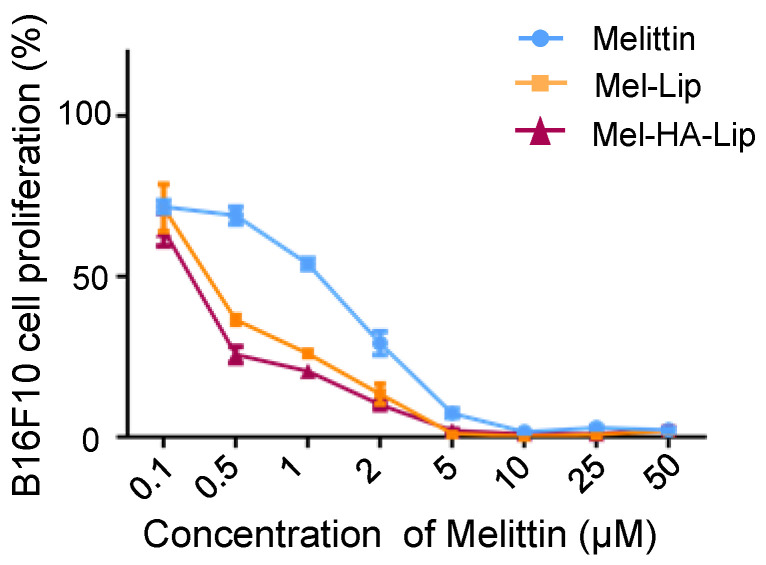
Cytotoxicity of B16F10 cells following treatment with free melittin (Melittin), Mel-Lip, and Mel-HA-Lip. (*n* = 3).

The cellular cytotoxicity of the blank and drug-loaded liposomes is shown in Figure 8 and Figure 9, respectively. In the experiments, the default initial drug concentration of the blank vectors was 1 mg/mL, consistent with the drug-loaded vectors. Blank vectors (Lip and HA-Lip) conferred little cytotoxicity to B16F10 cells within the range of 0.1–50 μmol/L, and the survival rate of B16F10 cells exceeded 90%. The cytotoxicity of Mel-Lip and Mel-HA-Lip was higher than that of the free drug group (*p* < 0.05) within the range of melittin concentration from 0.5 to 5 μM, while Mel-HA-Lip showed stronger cytotoxicity than Mel-Lip (*p* < 0.05) in the range of melittin concentration from 0.1 to 2 μM, consistent with the cellular uptake.

## 4. Discussion

In this study, we designed a new drug-delivery platform for melittin-loaded liposomes in order to reduce side effects, enhance its potency, and expand the clinical application of melittin.

Reverse-phase evaporation can be used to prepare Mel-Lip with a smaller particle size, PDI, and higher absolute zeta potential value and EE. This is because water-soluble drugs are usually encapsulated in the internal aqueous phase of liposomes, while the volume of the inner phase is important for the EE of liposomes. The liposomes prepared by the reverse-phase evaporation method had a larger internal water phase with good stability [32].

Importantly, the concentration of phospholipid and the ratio of drug to lipid played key roles in liposome preparation; thus, these two factors were further investigated in prescriptions using uniform design. HSPC and DSPE-PEG2000 were applied to construct composite phospholipid liposomes (two different phospholipids with different phase-transition temperatures are used as membrane materials). Liposomes with higher stability and bioavailability can be screened by assessing the effects of different phospholipid ratios on the rigidity of the liposomes. DSPE-PEG2000 modified nanocarriers have been widely applied. The hydrophobic end of DSPE-PEG2000 is inserted into the phospholipid membrane, while the hydrophilic end is placed on the surface of the nanocarriers to form a thin hydrogel layer, which prolongs the circulation time of the preparation in vivo [33]. Furthermore, by introducing a lipophilic group into HA, the hydrophobic part was inserted into the phospholipid bilayer during the phospholipid membrane hydration stage, which resulted in a smooth connection between HA and the liposome surface. After modifying liposomes with HA-DOPE, the liposome particle size was slightly smaller but with no significant difference (*p* > 0.05). Additionally, each disaccharide unit of the HA molecule contains a carboxyl group, which dissociates into anions under physiological conditions and contributes to the negative charge on the surface of the HA-modified nanovesicle; this could explain the improved absolute value for zeta potential with HA modification. Furthermore, the larger zeta potential produces stronger mutual repulsion between the nanocarriers, thereby enhancing the stability of the dispersion.

Based on the in vitro release behavior, HA-DOPE was inserted into the lipid bilayer to form a hydrogel network structure on the surface of the nanoparticles, which prolonged the circulation time of liposomes in vivo and played a protective and sustained release role. After 12 h, the HA layer began to be lost, accelerating the release rate of preparation, such that the rate of Mel-HA-Lip release gradually synchronized with that of the unmodified liposome. Moreover, the cumulative release rates were close to 100% after 48 h in both cases, indicating that the preparation had good bioavailability.

HA-modified nanocarriers can be transported into cells via receptor-mediated endocytosis, which is an active targeting and specific cellular uptake mechanism, selectively binding receptors on the cell membrane surface during internalization [34]. Thus, the HA ligand can selectively bind to the CD44 receptor on the surface of tumor cells and enhance internalization through active endocytosis [35]. B16F10 cells have been reported to express high levels of the CD44 receptor [36,37]; therefore, the HA modification significantly enhances the cellular uptake of liposomes. As the HA concentration increased, the targeting ability also improved. However, the uptake efficiency decreased when the concentration of HA was 1 mg/mL, possibly because of competition inhibition of free HA and HA-modified liposomes during binding to CD44 receptors. In this case, cellular uptake was dominated by passive-targeting endocytosis. In addition, the free HA ligand binds to CD44 on the cell surface, which may form a hydrophilic barrier and inhibit cellular penetration of preparations.

Blank liposomes had low cytotoxicity, suggesting that the preparation has favorable biological safety. The cytotoxicity of Mel-HA-Lip was significantly greater than that of Mel-Lip, and the cytotoxicity of both nanovesicles was higher than that of free drug, suggesting that the constructed liposome delivery system was able to enhance the anti-tumor activity. This is perhaps due to the free drugs penetrating tumor cells and accumulating by passive diffusion, which is limited by the drug concentration gradient within and outside the cell membrane and is also constrained by efflux pumps. In comparison, liposomes with membrane-like structures have compatibility with the cell membrane and endocytosis, leading to increased drug accumulation and enhanced cytotoxicity against tumor cells. In addition, the liposomes with HA modification exerted stronger cytotoxicity, indicating that HA modification can effectively deliver drugs to cancer cells, enabling drugs to accumulate and enhance cancer cell killing.

Based on HA-modified liposomes, this study successfully designed a melittin-loaded active targeting drug-delivery system and conducted a preliminary evaluation in vitro. The effect on tumor cell apoptosis, safety, and pharmacodynamics requires further verification; these will be the focus of follow-up work.

## 5. Conclusions

This current work demonstrated that both the particle size and PDI of Mel-HA-Lip decreased and the zeta potential increased compared with those of normal liposomes, which enhanced the stability of the preparation. Notably, the release of melittin from Mel-HA-Lip was sustained. In conclusion, HA-modified liposomes were able to specifically deliver the drug to melanomas and strengthen therapeutic action and improved the bioavailability and stability of the preparation. Melittin has been reported to have potential against melanoma [38]. Thus, our results suggest that the new targeted drug delivery system is a promising strategy for the treatment of melanoma. Our research methods and approaches lay a solid foundation for follow-up studies.

## Figures and Tables

**Table 1:** Uniform design for the selection of a Mel-Lip formulation. (*n* = 3).

Run	Factor		Mean Size (nm)	PDI	EE (%)	DL (%)
	x_1_ (mg/mL)	x_2_ (*w*/*w*)				
1	0.5	1:15	126.2	0.19	86.01	4.11
2	1	1:25	136.4	0.21	91.86	2.00
3	1.5	1:10	125.4	0.21	77.89	4.62
4	2	1:20	135.9	0.21	92.18	2.64
5	2.5	1:30	152.8	0.22	96.92	1.70

Note: x_1_, melittin concentration; x_2_, drug to lipid ratio.

**Table 2:** Characterization of Mel-Lip and Mel-HA-Lip. (*n* = 3).

Formulation	Mean Size (nm)	PDI	Zeta Potential (mV)	EE (%)	DL (%)
Mel-Lip	132.7 ± 1.55	0.23 ± 0.01	−11.5 ± 1.51	86.25 ± 1.28	3.91 ± 0.49
Mel-HA-Lip	124.3 ± 8.93	0.19 ± 0.02 *	−12.4 ± 1.08	90.43 ± 0.41 **	4.46 ± 0.10

Note: compared with Mel-Lip, * *p* < 0.05, ** *p* < 0.01.

## Data Availability

The data presented in this study are available on request from the corresponding author.
